# The importance of artificial wetlands for birds: A case study from Cyprus

**DOI:** 10.1371/journal.pone.0197286

**Published:** 2018-05-10

**Authors:** Efthymia Giosa, Christos Mammides, Savvas Zotos

**Affiliations:** 1 Department of Biological Applications and Technologies, University of Ioannina, Ioannina, Greece; 2 Nature Conservation Unit, Frederick University, Nicosia, Cyprus; 3 Guangxi Key Laboratory of Forest Ecology and Conservation, College of Forestry, Guangxi University, Nanning, China; 4 School of Pure and Applied Sciences, Open University of Cyprus, Nicosia, Cyprus; 5 Terra Cypria—The Cyprus Conservation Foundation, Limassol, Cyprus; Consejo Superior de Investigaciones Cientificas, SPAIN

## Abstract

The degradation of natural wetlands has significant effects on the ecosystem services they provide and the biodiversity they sustain. Under certain conditions, these negative effects can be mitigated by the presence of artificial wetlands. However, the conservation value of artificial wetlands needs to be explored further. In addition, it is unclear how certain anthropogenic variables, such as road networks and hunting reserves (i.e., areas where hunting of birds is prohibited) affect biodiversity in both artificial and natural wetlands. Here, we use data from thirteen artificial and six natural wetlands in Cyprus, to assess their similarities in bird species diversity and composition, and to quantify the relationship between species diversity and the density of road networks, hunting reserves, wetland size, and wetland depth. We found that while on average natural wetlands have more species and support higher abundances, certain artificial wetlands have the potential to support similarly diverse communities. Overall, regardless of the type, larger wetlands, with shallower waters tend to be more biodiverse. The same is true for wetlands surrounded by a higher percentage of hunting reserves and a lower density of road networks, albeit the effect of road networks was weaker. We conclude, from our results, that although the conservation value of natural wetlands is higher, artificial wetlands have the potential to play a complimentary role in the conservation of bird communities, assuming those wetlands have the right characteristics (e.g., in terms of size and depth) and assuming that the disturbances resulting from high-impact human-activities (e.g., hunting) are minimized.

## Introduction

Historical and current rates of land modification have resulted in the loss of more than half of the wetlands worldwide [1,2]. This loss has affected many of the key ecosystem services that wetlands provide–such as water purification and control of sediment flow [3]–and has affected also the biodiversity that depends on wetlands for its persistence [2,4]. Wetlands are particularly important for waterbirds [5], which depend on them for finding food and breeding grounds. The degradation of wetlands can have significant conservation implications extending beyond the local scale when degradation affects migratory birds, which rely on wetlands for suitable habitat during the migration [6,7].

Efforts are being made worldwide, at the national and international level, to conserve wetlands of importance [8,9]. Perhaps the most notable effort is the Ramsar Convention on Wetlands [9], currently ratified by 169 countries. Until today, more than 2000 wetlands worldwide have been designated as Ramsar sites, covering an area of about 215 million ha. Many of those wetlands are artificial [2]. Artificial wetlands can vary in size and other biophysical characteristics, ranging from small agricultural ponds [10] and rice-paddy fields [11], to water treatment facilities [12] and large water reservoirs [13]. Previous studies have shown that although the construction of artificial wetlands can have negative environmental effects [14,15]–e.g., when dams fragment river ecosystems–in some cases they also have the potential to play a crucial complementary role in conserving biodiversity [5,13,16–23] and in maintaining ecosystem services [24–27]. Having said that, it should be noted that artificial wetlands may provide less suitable habitat than natural or restored wetlands [1]. The conservation value of artificial wetlands is contingent on multiple factors. Ma et al. [1] reviewed the literature and identified several generalizable characteristics that can make artificial wetlands useful to biodiversity. For example, size, water depth, vegetation, topography, and wetland connectivity were all found to affect the presence of waterbirds [1].

Although generally it is well understood which biophysical characteristics of wetlands have the potential to contribute to more diverse communities [1,4,12,25,28], there still remain gaps in our knowledge regarding the conservation value of wetlands worldwide. Firstly, more studies are needed juxtaposing the species communities of comparable types of artificial and natural wetlands [29]. Without such assessments, the relative conservation importance of artificial wetlands remains ambiguous. Secondly, unlike biophysical characteristics, it is unclear how certain anthropogenic factors effect biodiversity in artificial, but also natural wetlands–especially at the landscape level [30]. In this study, we used data from thirteen artificial and six natural wetlands in Cyprus to: a) assess whether bird diversity and composition differ between the two types of wetlands, and b) identify which anthropogenic variables influence bird species richness and diversity in wetlands. Specifically, we measured the relationship between species richness and diversity and nearby road networks and hunting reserves (i.e., areas in which the hunting of birds is prohibited). We included wetland size and depth as control variables, as they have been shown to be strong determinants of species diversity in wetlands [1].

Larger wetlands have the potential to support more species as they usually have higher habitat heterogeneity and therefore more breeding and foraging grounds [1,31]. Similarly, water depth is important [31] because it affects habitat accessibility; shallower wetlands tend to have more species because they are more suitable to a wider range of non-diving waterbirds, which cannot forage in deep waters [1,28]. Roads have been shown to affect negatively most bird communities [32,33], and therefore we hypothesized that they will be associated with reduced diversity at our study sites. For example, previous research has shown that roads fragment habitats, limit movement, increase mortality, and that traffic noise affects the behaviour and reproductive success of birds [34–36]. However, the effects of roads have been mainly assessed on non-wetland species. Yet, many biodiverse wetlands have roads with high-traffic flow in the neighbouring area, especially in countries with dense road networks, such as Cyprus [37]. It is therefore important to study in more detail whether wetland species are equally vulnerable to this anthropogenic threat.

Similarly, the relationship between hunting reserves and waterbird species diversity needs to be assessed further. Although the effects of hunting on waterbirds have been studied [38], this was done mostly by exploring the impact caused by hunters [39] or by assessing the consequences resulting from lead poisoning [40–42], and less often by assessing the effectiveness of the hunting reserves themselves (but see [43,44]). Hunting is a popular activity in our study area, which causes direct mortality and indirect disturbance, therefore we hypothesized that the extent of hunting reserves will be positively related to bird diversity.

## Materials and methods

### Study area and sites

Cyprus is the third largest island in the Mediterranean Sea, covering an area of approximately 9,000 km^2^ [45]. It is recognized as an important area for birds, with over 400 species recorded [46]. About one-third of the species are resident, while the rest are either regular or occasional migrants [45,46]. Millions of birds use the island’s habitats as stopover sites or wintering grounds during their migrations between Europe and Africa in the autumn and the spring [46]. Many of those birds, some of which are of European and global importance, rely on the island’s wetlands for their persistence. Due to the island’s Mediterranean climate, and consequently the occasionally limited rainfall, water resources often become scarce and insufficient to cover the domestic and agricultural needs of the local human population. As a response, during the last few decades, a dense network of irrigation reservoirs and dams have been built to address this issue [47]. Some of these are recognized today as important areas for biodiversity, especially for waterbirds, complementing the small number of natural wetlands found on the island.

**Fig 1:**
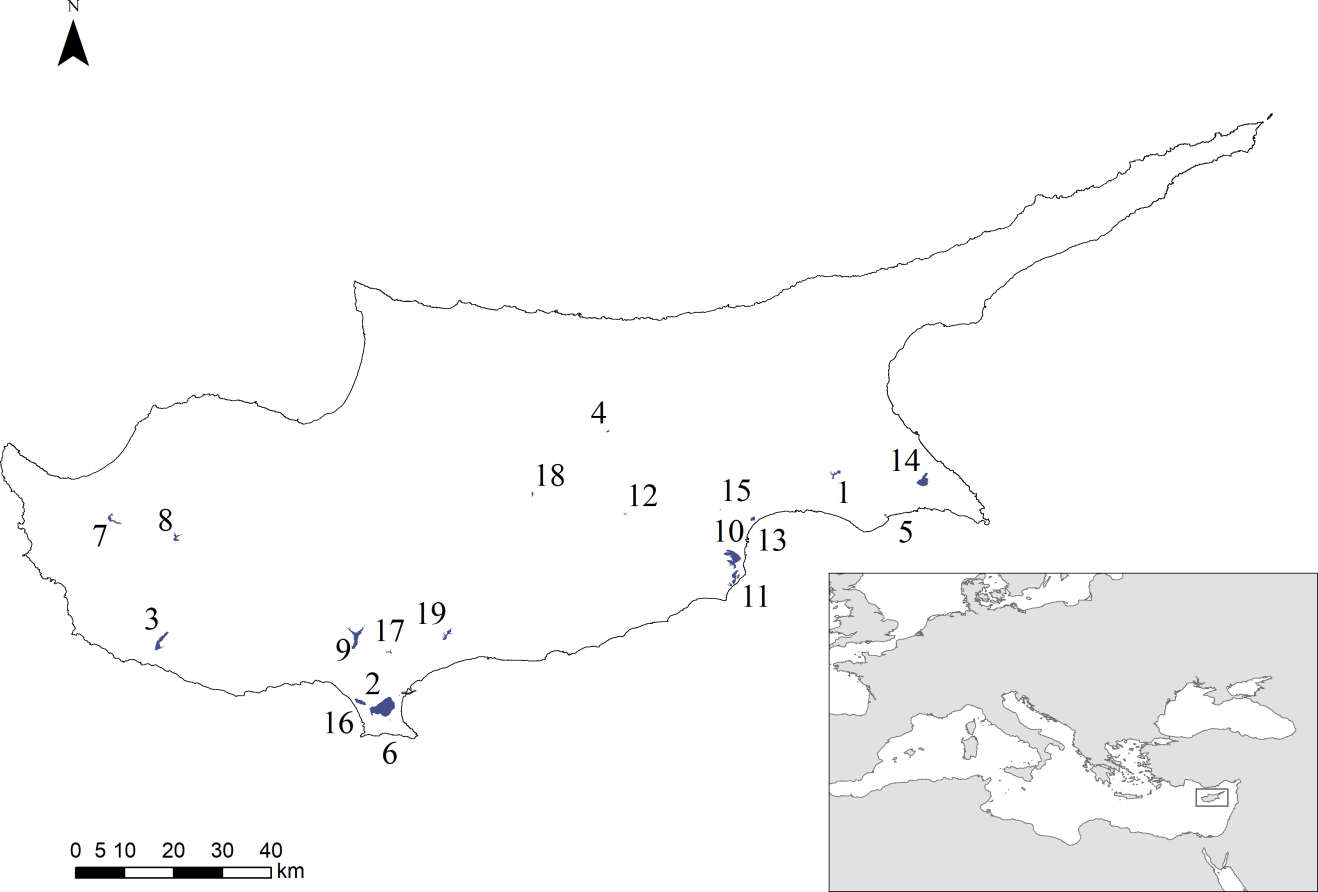
A map of Cyprus showing the location of the nineteen study sites. The numbers correspond to those in Table 1. The inset map on the bottom right corner shows Cyprus’ location in the eastern Mediterranean Sea (source: 2009–2017 Natural Earth). The island’s location makes it an important stopover site from millions of migratory birds.

As part of its wetland monitoring program, BirdLife Cyprus surveys every month the bird communities at multiple artificial and natural wetlands. For the purposes of this study, we used the data from the surveys of 2009, 2010, and 2011 –collected monthly between January and December–covering nineteen wetlands (Fig 1). Those consisted of eleven dams, one irrigation pond, one water treatment facility, two natural salt lakes, three marshes, and one coastal wetland (Table 1). Eight out of the nineteen sites are within the boundaries of areas classified as Important Bird Areas (IBAs), as defined by BirdLife International; three of those sites are artificial reservoirs [46]. The two salt lakes are the island’s two Ramsar sites.

**Table 1:** List of the wetlands included in the study, along with a description of their type (0 = Artificial, 1 = Natural) and their cumulative bird species richness (S) in 2009, 2010, and 2011. Please note that site 5 (Ayia Thekla–Liopetri) was only surveyed in 2010 and 2011.

Site	Name	Description	Type	S_2009_	S_2010_	S_2011_
1	Akhna Dam	Dam	0	61	62	55
2	Akrotiri Salt Lake/Zakaki Pond	Salt Lake	1	87	81	89
3	Asprokremnos Dam and Pools	Dam and Irrigation Ponds	0	50	47	46
4	Athalassa Dam	Dam	0	23	24	21
5	Ayia Thekla—Liopetri River	Coastal Wetland	1	NA	4	13
6	Bishop’s Pool	Irrigation Pond	0	50	45	50
7	Evretou Dam	Dam	0	24	33	30
8	Kannaviou Dam	Dam	0	16	17	13
9	Kourris Dam	Dam	0	15	15	11
10	Larnaca Salt Lake	Salt Lake	1	69	71	69
11	Larnaca Sewage Works	Water Treatment Facility	0	70	63	57
12	Lymbia Dam	Dam	0	3	9	14
13	Oroklini Lake	Marsh	1	57	52	63
14	Paralimni Lake	Marsh	1	31	39	42
15	Partenitis Dam	Dam	0	30	30	26
16	Phassouri Reedbed	Marsh	1	24	23	46
17	Polemidia Dam	Dam	0	6	12	10
18	Tamassos Dam	Dam	0	14	5	4
19	Yermasoyia Dam	Dam	0	20	18	21

### Data collection

#### Bird surveys

To estimate bird diversity at each wetland we extracted from BirdLife’s monthly reports of 2009, 2010, and 2011 the list of species sighted between January and December, along with the number of individuals for each species. Apart from the coastal wetland (Ayia Thekla–Liopetri) that was only surveyed in 2010 and 2011 (Table 1), the rest of the wetlands were surveyed during all three years. Wetlands were surveyed concurrently, once a month (around the 20^th^), by a small team of experienced volunteers. At each wetland, the volunteers counted all the birds present. Each survey lasted between one to six hours depending on the size of the wetland and the number of birds present (Martin Hellicar, Director of BirdLife Cyprus, personal communication).

Using the monthly bird surveys, for each year and each wetland we calculated: a) the cumulative species richness, by counting the total number of unique species recorded during that year, and b) the species diversity, using the Fisher’s alpha diversity index [48]. The index is used commonly because of its efficiency in discriminating between sites [49]. It was considered appropriate for our data because it assumes a log-series distribution and it is influenced less by the few extremely abundant species [49]. In addition to estimating species richness and diversity, we used the IUCN Red List [50] to record each bird’s IUCN status, in order to assess how species of conservation importance were distributed between the wetlands.

#### Size, depth, and anthropogenic variables

We measured the size of each wetland (in km^2^) using ArcMap (v. 10.2) and the spatial map provided by the Water Development Department (WDD) of the Government of Cyprus, which marked the boundaries of the wetlands. WDD also provided us with data on the depth and the salinity of each wetland. The data on the depth represented the maximum depth of each wetland in meters. For the purposes of the analysis, we classified wetlands into shallow (≤ 10 m) and deep (> 10 m). The data on salinity categorized wetlands into four categories: freshwater (<5 practical salinity units (psu)), brackish (5–30 psu), saline (30–50 psu), and hypersaline (>50 psu). We did not use salinity as an explanatory variable in our analyses because it correlated highly with depth: all non-freshwater wetlands were shallow and all but two freshwater wetlands were deep. We should also note that all thirteen artificial wetlands belonged to the freshwater category. There were four brackish natural wetlands, one saline, and one hypersaline.

To estimate the density of the road network at each site, we used ArcMap to create a “buffer” of 250 m around each wetland, within which we measured the total length of roads (in km), using the road map of the island provided by the Department of Land Surveys of the Government of Cyprus. We then divided the total road length by the area of the buffer to calculate the corresponding road density (in km/km^2^). We chose this buffer size because we wanted to capture effectively the area adjacent to the wetlands that is used by the birds as feeding and nesting grounds. In addition, past research on birds has shown that landscape characteristics are most relevant within a radius of 125 to 250 m [51]. Within that same buffer, we calculated the percentage of area cover by hunting reserves, by georeferencing the official hunting map of 2011, released by the Game Fund Service (i.e., the governmental authority responsible for managing the wild avifauna in Cyprus and for regulating hunting). The reserves are essentially areas of various sizes and habitats, spread throughout the island, in which the hunting of birds is prohibited.

### Data analysis

#### Differences in bird diversity and composition

For all the statistical analyses we used the R programming language [52] and considered results statistically significant if p-value was less the 0.05. For this part of the study, in which we assess whether bird diversity and composition differ between the two types of wetlands, we ran each of the analyses–described below–separately for each year. In this way, we were able to evaluate the robustness of the results. To test whether annual species richness and Fisher’s alpha diversity differed between natural and artificial wetlands we used two-tailed t-tests. To assess whether the annual composition of the bird communities differed between the two types of wetlands we ran an analysis of similarities test (ANOSIM), using the *anosim* function in the “vegan” package [53]. We ran the test twice for each year: first using only presence/absence data, and then using species abundances as well. In both cases, we first calculated the Bray-Curtis dissimilarity matrix and then ran *anosim* using 999 permutations. To visualize the dissimilarities in species composition for each year we used the non-metric multidimensional scaling (NMDS) ordination method. Through NMDS, we explored whether wetlands were clustered depending on their type. As before, we used the Bray-Curtis dissimilarity index. Calculations were carried out using the *metaMDS* function in the “vegan” package [53].

In addition, for the analysis based on the abundance data, we ran for each year a Similarity Percentages test (SIMPER), using the *simper* function in the “vegan” package”, to measure each species’ contribution to the Bray-Curtis dissimilarity matrix [53]. We then used the *indval* function in “labdsv” package [54] to measure each species’ relative average abundance in artificial vs. natural wetlands. In this way, we estimated for each species separately the proportion of the average abundance found in artificial wetlands as opposed to natural wetlands [54].

We also explored whether beta diversities within the two types of wetlands differed. For each of the three years, we measured the possible range of beta diversities within each type using the *beta*.*sample*.*abund* function in the “betapart” package [55]. The function samples a pre-specified number of wetlands each time (two in our case) and measures their “abundance-based dissimilarity” using the Bray-Curtis dissimilarity index [55]. By repeating the process 999 times, the full range of beta diversities within each type was obtained. Using those ranges, we calculated the probability that either type of wetlands had a higher beta diversity using the method described in the “betapart” package [55]. For comparison purposes, we also measured (using the same approach) the beta diversity when all wetlands were included regardless of the type. Finally, to evaluate whether the communities of the artificial wetlands were nested subsets of the communities found in natural wetlands, we used the *beta*.*pair* function in the “betapart” package to partition the pairwise beta diversity between each artificial wetland and natural wetland–as calculated using the Sorensen index [55]–into its turnover and nestedness components [56]. We then used the latter component to assess for each artificial wetland its degree of nestedness within each of the six natural wetlands for each of the three years separately.

#### Influence of size, depth, and anthropogenic variables

To quantify the relationship between the four explanatory variables (i.e., size, depth, road network density, and percentage of hunting reserves) and the species richness and diversity of wetlands, we used linear multiple regression analyses and selected models using the information-theory approach [57]. For this part of the analysis, we used only the bird data from 2011, since the data for the hunting reserves were from that year. For each of the two measures of bird diversity (i.e., species richness and Fisher’s alpha diversity) we first ran the full regression model, which included all four variables, and then, using the “MuMIn” package [58], we ran all possible subset models and ranked them based on their corrected Akaike Information Criterion (AICc) [57]. We selected as plausible models (i.e., models explaining the observed variation in the response variables) all the models with a ΔAICc of less than 2 [59]. We averaged the unstandardized and standardized regression coefficients of the selected models using the *model*.*avg* function in that same package. We used the zero-method to average both types of coefficients because it is more appropriate for studies such as ours in which one is interested in assessing the relative importance of each predictor [60].

To normalize our data we log-transformed area and road density. To ensure that our models did not violate the normality assumption of the linear regression method, we used the Shapiro-wilk normality test to assess the residuals of the two full models, using the *shapiro*.*test* function in R. We also used the Moran’s I test in the “ape” package [61] to assess whether the full models’ residuals were spatially autocorrelated. Lastly, we used the *vif* function in the “car” package [62] to measure the variance inflation factor of each explanatory variable to assess whether there was any collinearity.

## Results

In total, 233,144 birds were recorded belonging to 113 species. A few of those, such as the Common Kingfisher (*Alcedo atthis*), although not strictly waterbirds, are known to depend on water ecosystems for resources and therefore were kept in the analyses. The complete list of all the species, along with the number of individuals recorded in each year can be found online in the information made available on figshare [63]. The number of species recorded at each site did not vary substantially over the three years (Table 1); the Pearson’s pairwise correlations between species richness in 2009, 2010, and 2011 ranged from 0.94 to 0.98, showing that the number of species visiting each site showed little annual variation. Similarly, the corresponding correlations for Fisher’s alpha diversity ranged from 0.88 to 0.95.

Overall, 109 species were recorded in natural wetlands and 100 species in artificial; about 12% of the species were recorded only in natural wetlands and 4% only in artificial wetlands. According to the IUCN Red List, four of the recorded species were threatened: one endangered (EN) and three vulnerable (VU). One of the threatened species was found only in natural wetlands, one in both types, while two only in artificial wetlands. Although the abundance of the three of those threatened species was limited to a few individuals only (2–4), in the case of the Common Pochard (*Aythya ferina*), which is listed as vulnerable, a total of 241 individuals were recorded with almost 75% of them in artificial wetlands. Ten other species were near threatened (NT); two of those were found only in natural wetlands, while eight in both types. The complete list of the species recorded along with their IUCN status, abundances in artificial and natural wetlands, and abundances in years 2009, 2010, and 2011 are available online on figshare [63]

The average area of the wetlands was 1.75 km^2^ (*median* = 0.91), ranging from 0.02 to 10.07 km^2^, while the average maximum depth was 27 m (*median* = 14, *range* = 1–110). The average road density was 2.17 km/km^2^ (*median* = 2.06, *range* = 0.31–6.59), while the average area covered by hunting reserves was 82% (*median* = 85, *range* = 25–100). The complete data for each wetland are also available online, on figshare, along with the GPS coordinates and other descriptive information [63].

### Differences in species diversity and composition

According to the results of the t-tests, we did not detect any statistically significant differences between the species richness and the alpha diversity of the two types of wetlands for any of the three years examined (*p-values* for species richness ranged between 0.061 and 0.265 and *p-values* for Fisher’s alpha diversity ranged between 0.053 and 0.304; S1 Table). When the difference between the composition of the bird communities in natural and artificial wetlands was examined–using ANOSIM and only the presence/absence data–the results for year 2010 were statistically significant (*p-value =* 0.041), but not for years 2009 and 2011 (*p-values* = 0.089 and 0.137 respectively). However, when abundances were added the differences in species composition became statistically significant for all three years (*p-values* for 2009, 2010 and 2011 = 0.031, 0.005, 0.005 respectively).

**Fig 2:**
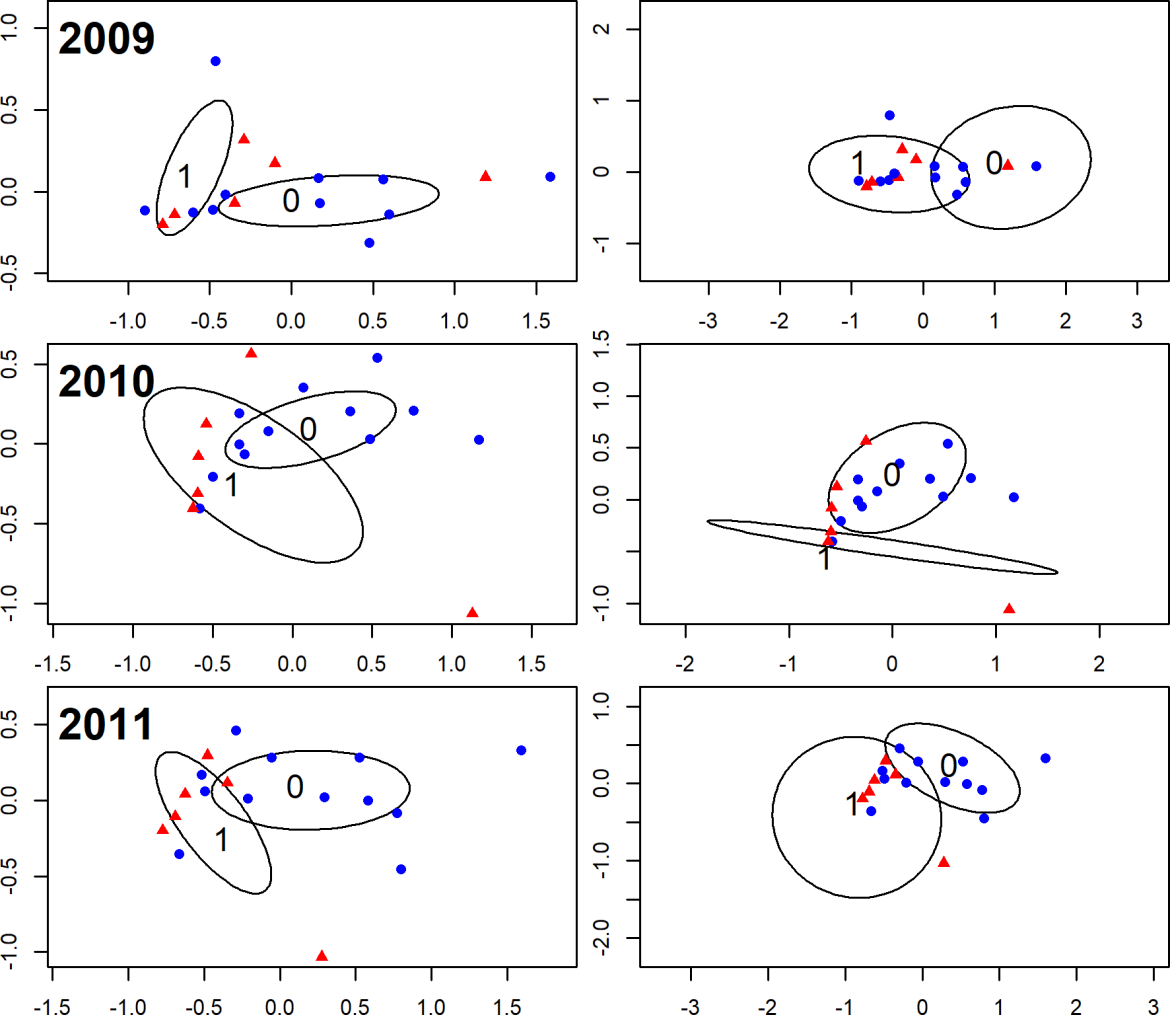
Results of the NMDS analyses for the three years, showing the dissimilarity in species composition between artificial and natural wetlands, based on the Bray-Curtis dissimilarity index, when presence/absence (left column) and abundances (right column) are used. Blue circles (●) correspond to artificial wetlands and red triangles (▲) to natural wetlands.

In particular, the NMDS analyses showed that in general sites were clustered according to their wetland type although there was a slight overlap between the two groups and certain artificial wetlands were consistently placed near natural wetlands (Fig 2). Based on the SIMPER results, the dissimilarities between the two types of wetlands were mostly due to 23–25 species (depending on the year examined), which combined accounted for 90% of the differences (S2 and S3 Tables). For example, the species with the highest contribution to dissimilarity in all three years was the Greater Flamingo (*Phoenicopterus roseus*), which had several thousands of individuals but was found mostly in the two salt lakes.

Although most of the differences were due to approximately a quarter of the species (Table 2), most species had a lower average abundance in artificial wetlands compared to natural wetlands. For instance, in 2011, the relative average abundance of approximately 75% of the species was less than 40% in artificial wetlands. The corresponding value for the years 2009 and 2010 was 72% and 59% respectively. These results mean that for most of the species, natural wetlands supported consistently more than half of their average abundance.

**Table 2:** The list of species accounting for 90% of the dissimilarity in species composition between artificial and natural wetlands in 2011, when abundances are used. For each species, the average and the relative average abundances in each wetland type are shown, along with the cumulative contribution to the Bray-Curtis dissimilarity index.

Species	Average abundance		Relative average abundance		Cumulative Contribution
	Artificial	Natural	Artificial	Natural	
*Phoenicopterus roseus*	0.00	7044.67	0.00	1.00	0.23
*Fulica atra*	441.46	485.33	0.48	0.52	0.35
*Charadrius alexandrinus*	7.15	550.00	0.01	0.99	0.41
*Anas crecca*	102.92	467.67	0.18	0.82	0.47
*Tachybaptus ruficollis*	186.38	99.67	0.65	0.35	0.51
*Anas platyrhynchos*	236.85	182.33	0.57	0.43	0.55
*Spatula clypeata*	144.62	432.50	0.25	0.75	0.59
*Larus ridibundus*	37.77	730.17	0.05	0.95	0.62
*Gallinula chloropus*	69.00	96.17	0.42	0.58	0.64
*Bubulcus ibis*	102.69	18.17	0.85	0.15	0.67
*Larus cachinnans*	64.31	131.33	0.33	0.67	0.69
*Vanellus vanellus*	7.69	60.17	0.11	0.89	0.71
*Vanellus spinosus*	55.38	63.17	0.47	0.53	0.74
*Calidris minuta*	6.77	336.83	0.02	0.98	0.76
*Himantopus himantopus*	13.23	150.50	0.08	0.92	0.77
*Phalacrocorax carbo*	39.08	4.67	0.89	0.11	0.79
*Charadrius leschenaultii*	1.23	37.33	0.03	0.97	0.80
*Plegadis falcinellus*	10.92	70.83	0.13	0.87	0.82
*Tringa glareola*	12.23	58.50	0.17	0.83	0.83
*Egretta garzetta*	18.92	84.67	0.18	0.82	0.85
*Glareola pratincola*	2.15	31.50	0.06	0.94	0.86
*Tadorna tadorna*	0.15	305.33	0.00	1.00	0.87
*Ardea cinerea*	21.54	144.00	0.13	0.87	0.88
*Calidris pugnax*	8.15	89.50	0.08	0.92	0.89

**Fig 3:**
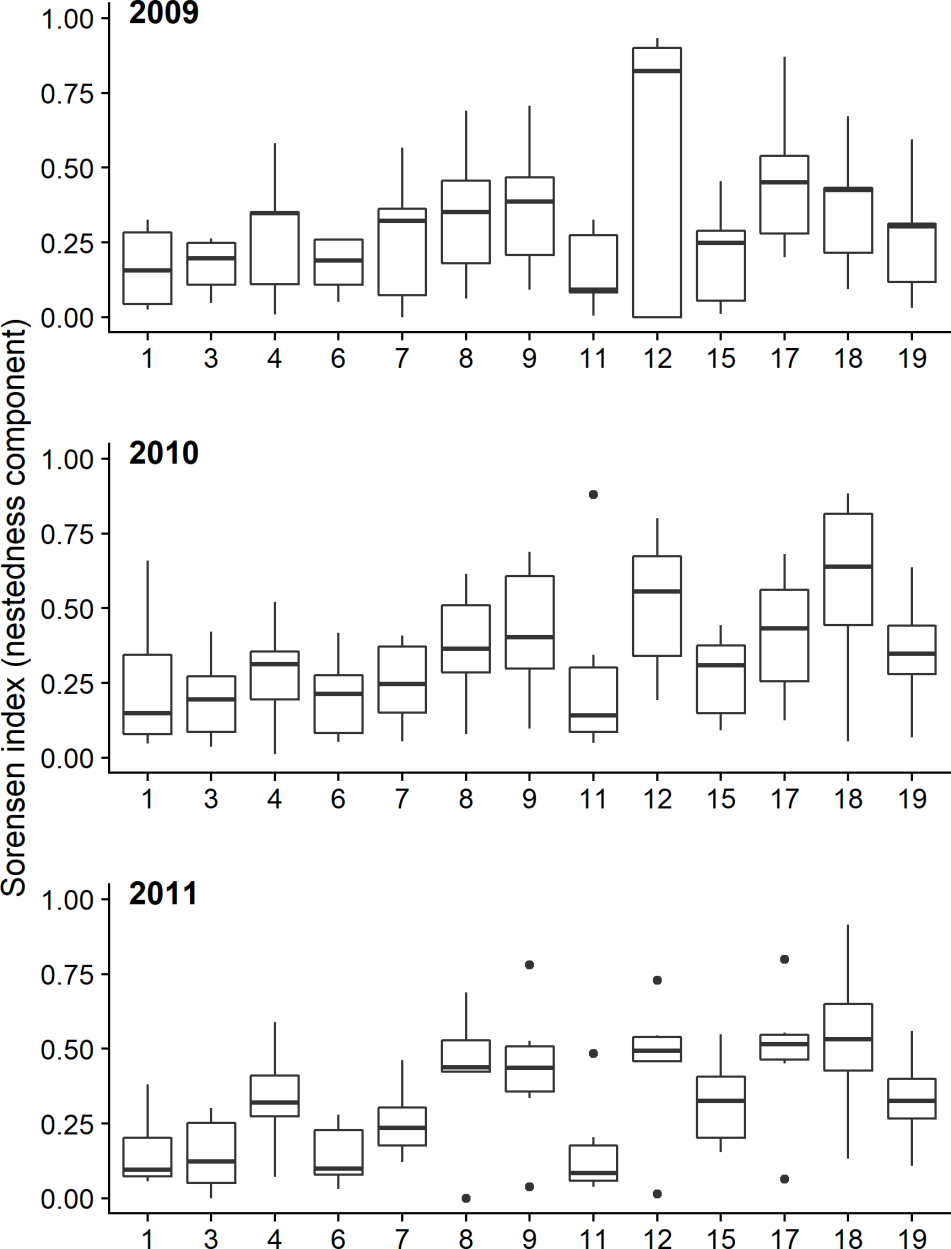
Boxplots showing the extent to which bird communities in each of the thirteen artificial wetlands are nested subsets of the communities in natural wetlands, as measured using the Sorensen index for each of the three years separately. The numbers on the x-axis correspond to the numbers of the artificial wetlands in Table 1. Each boxplot is made using six data points, representing the nestedness between each artificial wetland and the six natural wetlands. The higher the value the higher the degree of nestedness. Boxplots are based on the median and the 1^st^ and 3^rd^ quartiles. Outliers represent the points that extend beyond 1.5 times the interquartile range.

The analysis of the beta diversities–measuring how biodiverse the wetlands are within each type–showed that the difference between the two was not statistically significant, regardless of the year examined (*p-values* for 2009, 2010 and 2011 were 0.468, 0.584, and 0.436 respectively; S1 Fig). When nestedness was analysed, results showed that some of the bird communities in artificial wetlands, such as those in Tamassos and Polemidia Dams, are mostly subsets of the communities found in natural wetlands (Fig 3). However, some others, such as those in Akhna Dam and Asprokkremos Dam & Pools, had low nestedness values (e.g., <0.25), suggesting that they are relatively different from those recorded in natural wetlands. These patterns were consistent across all three years (Fig 3)

### Importance of size, depth, and anthropogenic variables

The adjusted R^2^ values of the two linear multiple regression models, with which we measured the relationship between the four explanatory variables and species richness and diversity, were 0.70 and 0.42 respectively. The values suggest that the selected variables captured most of the observed variance in species richness and much in diversity. There were no issues, in either case, with normality (Shapiro-Wilk normality test *p-values* = 0.80 and 0.16), spatial autocorrelation (Moran’s I *p-values* = 0.63 and 0.52), or collinearity (highest variance inflation factor was 1.16). There were two models with a ΔAICc of less than 2 for species richness and six models for species diversity (S4 Table).

**Fig 4:**
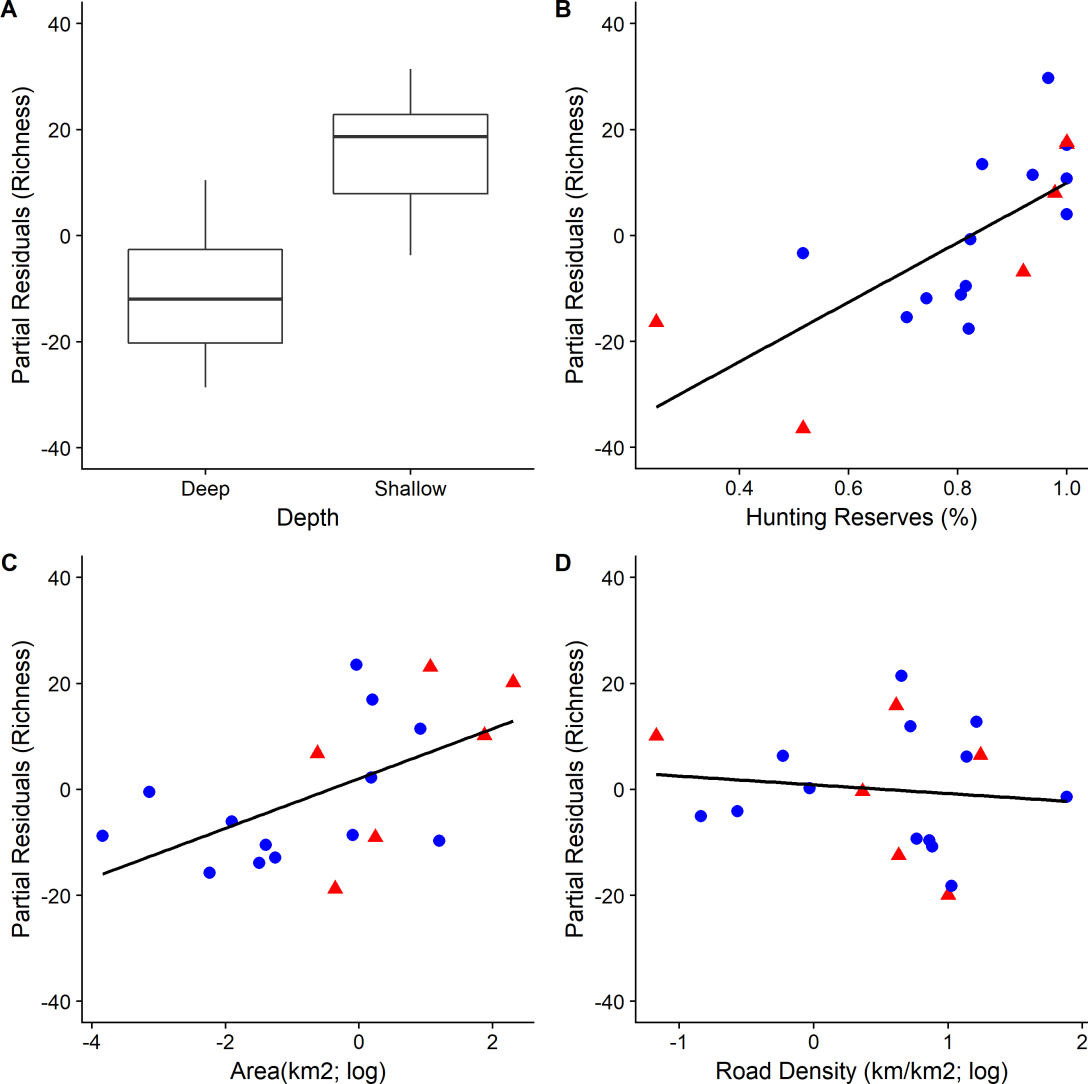
Partial residual plots illustrating the relationship between each of the four explanatory variables and species richness (S) in year 2011. Blue circles (●) correspond to artificial wetlands and red triangles (▲) to natural wetlands. Boxplots are based on the median and the 1^st^ and 3^rd^ quartiles.

**Fig 5:**
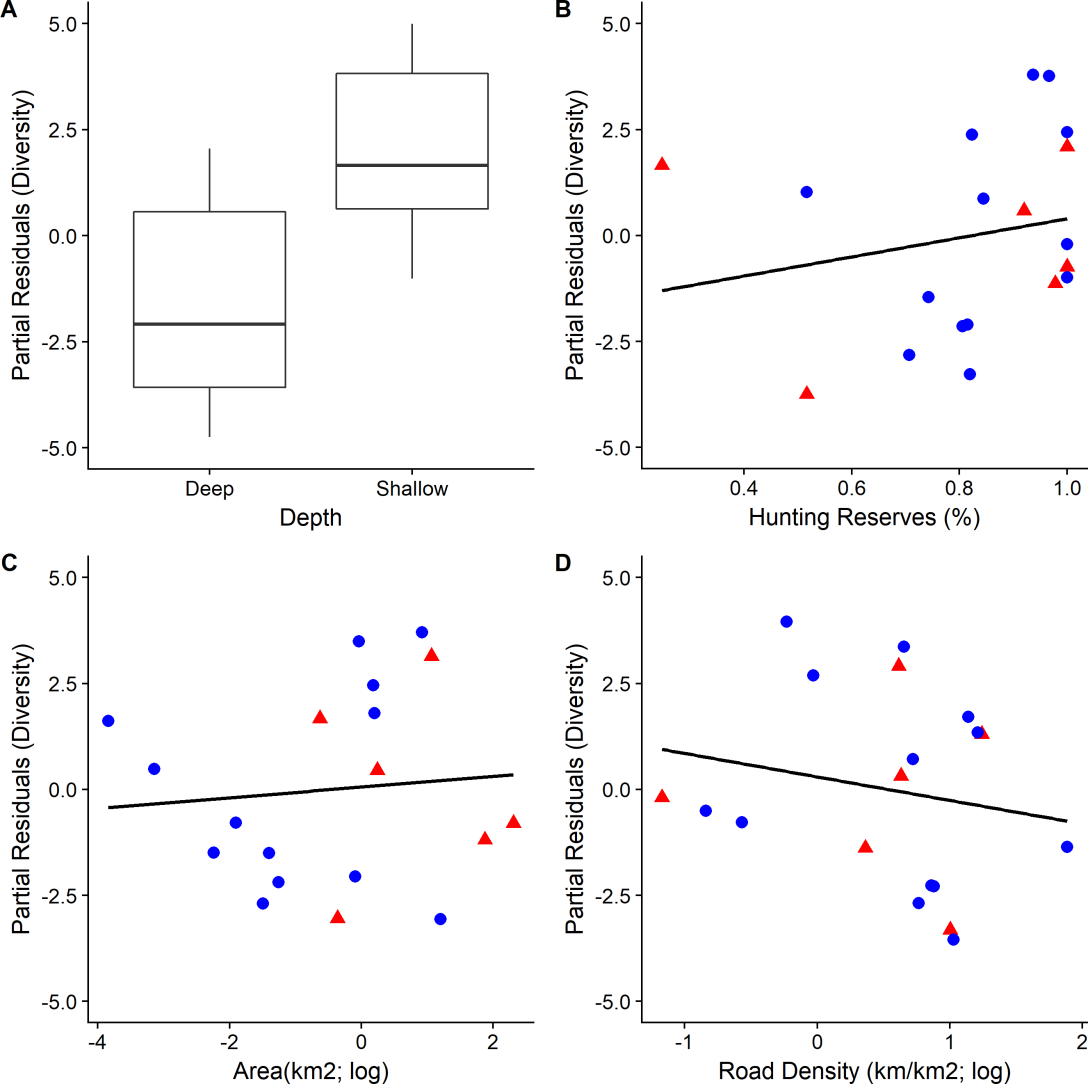
Partial residual plots illustrating the relationship between each of the four explanatory variables and species diversity (Fisher’s alpha diversity) in year 2011. Blue circles (●) correspond to artificial wetlands and red triangles (▲) to natural wetlands. Boxplots are based on the median and the 1^st^ and 3^rd^ quartiles.

The depth of the wetlands was the most important factor, both for species richness and diversity, and had the largest relative effect as indicated by the standardized regression coefficients (0.57 and 0.53 respectively; Table 3 and Figs 4 and 5). Shallower wetlands tended to have more diverse communities. The percentage of area covered by hunting reserves, was the second most important variable for species richness (0.48, *RI* = 1.00, Table 3); wetlands surrounded by more hunting reserves had more species (Fig 4). The relationship between the reserves and the species diversity was also positive (0.14) but weaker (Fig 5). The size of the wetlands was also important, for both richness and diversity, albeit less than the depth and the hunting reserves. Wetlands surrounded by higher road density had lower diversity, although the relationship was not as strong for species richness (Table 3).

**Table 3:** Results of the linear multiple regression analyses showing the averaged standardized (beta) and unstandardized (β) regression coefficients for each of the four explanatory variables, along with their relative importance (RI).

Variable	Species Richness			Species Diversity		
	beta	β	RI	beta	β	RI
Depth (deep vs shallow)	0.57	26.67	1.00	0.53	3.52	1.00
Hunting reserves (%)	0.48	56.22	1.00	0.14	2.26	0.44
Area (km^2^; log)	0.32	4.70	1.00	0.06	0.12	0.22
Road Density (km/km^2^; log)	-0.05	-1.65	0.28	-0.13	-0.55	0.39

## Discussion

On average, natural wetlands had higher waterbird diversity than artificial wetlands. The results of our analyses though suggest that the differences in species richness and diversity between the two types of wetlands were not statistically significant for any of the three years examined, although the differences in species composition, especially once abundance was incorporated, were significant. Based on these findings, it can be argued that some of the artificial wetlands in our dataset, such as the water treatment facility in Larnaca and Akhna Dam (Table 1), have the potential to support similarly diverse bird communities as natural wetlands. The results of the regression models suggest that wetland characteristics, such as size and depth, may be more important in determining species richness and diversity than the type itself. Apart from a small number of species that are specialists and tend to depend on particular types of habitats, which cannot be readily provided by artificial reservoirs, most other species (about 88%) were found in both types of wetlands.

It should be clarified though that the relative average abundance of most of the species was lower in artificial wetlands compared to natural wetlands. Although the results of the SIMPER analysis showed that 90% of the differences in species composition could be attributed to a quarter of the species only, the analysis of the relative average abundances showed that artificial wetlands supported less than 40% of the average abundance of the majority of the species. Previous research has also shown that artificial wetlands have a lower conservation value when abundances are considered [29,64]. Consequently, artificial wetlands may not be as important as natural wetlands for conserving species, but they can have a valuable complementary role in maintaining beta diversity in species-rich areas [5,65].

The potential value of the artificial wetlands can be particularly important when migratory birds of international importance are considered. Such species could depend on these areas for appropriate habitat during their migration, making artificial wetlands valuable at an international level. This idea is supported by our results, which showed that out of the fourteen near-threatened and threatened species, eleven of them were also found in artificial wetlands, with two of them, the endangered White-headed Duck (*Oxyura leucocephala*) and the vulnerable Marbled duck (*Marmaronetta angustirostris*) found only in artificial wetlands [63]. It should be noted though that even within the artificial wetlands, only two individuals from each of these two species were recorded. Therefore, this pattern should be interpreted cautiously and not necessarily as preference of the two species towards artificial wetlands. Even so, this does not negate the fact that certain artificial wetlands seem capable of providing refuge to species of conservation importance and can support diverse communities.

Not all artificial wetlands, however, are or can be equally biodiverse. One of the wetlands in our dataset for example, Tamassos dam, had only four species in 2011 and five in 2010, and had expectedly high pairwise nestedness values with natural wetlands (Fig 3). The artificial wetland that consistently had the highest number of species during all three years (*n* = 63–70 species) was the reservoir of the water treatment facility in Larnaca, which is located very close to the Larnaca Salt Lake, the second most biodiverse wetland (*n* = 69–71), following the Akrotiri Salt Lake (*n* = 81–89). The proximity of the reservoir to the salt lake is likely to be one of the key factors determining its high species richness. Yet, the second most biodiverse artificial wetland was the Akhna Dam (*n* = 55–61), which is not located next to any natural wetland, suggesting that artificial wetlands have the potential to contribute to the persistence of numerous species on their own, and that other factors are also important in determining wetlands’ species diversity.

For example, our results suggest that wetland depth and size are likely to be major determinants of bird species richness and diversity, a finding in agreement with the results of previous studies [6,12,66,67]. Nonetheless, the importance of depth in our study should be interpreted cautiously because as mentioned depth covaried with salinity and potentially other factors too, such as vegetation type (that itself may be determined both by depth and by salinity). Consequently, the relationship between depth and species richness and diversity may be reflecting the importance of these other variables [1] as well, overestimating the significance of depth. Salinity, for example, is known to have a strong effect on bird species richness [1,68,69], but unfortunately we could not test it independently.

Besides depth, our analysis showed that another important factor is the percentage of the area covered by reserves where hunting is prohibited. We acknowledge that this is not necessarily a cause and effect relationship. It is possible that the hunting prohibition has no impact, and that the large regression coefficient (Table 3) reflects the possibility that the authorities have banned hunting near wetlands with many species. However, the bivariate Pearson’s correlation coefficient between the percentage of hunting reserves and species richness and diversity was 0.47 and 0.34 respectively, suggesting that there are biodiverse wetlands with relatively low percentages of reserves (e.g., Paralimni Lake) and vice versa (e.g., Athalassa Dam). Considering the high number of hunters in the island and the fact that multiple of the species recorded are game species, such as the Greylag goose (*Anser anser*) and the Greater white-fronted goose (*Anser albifrons*), it is highly likely that the imposed prohibition has a positive effect on the number of species present.

This finding matches the results of a previous study, in which the determinants of the species richness of birds, in forty-eight protected areas in Cyprus, were assessed [70]. It was found that compared to other bird groups wetland birds benefited more from the presence of hunting reserves in protected areas [70]. Hunting can affect waterbirds in multiple ways. In addition to increasing the direct mortality of game species [39], it can alter birds’ behaviour and result in reduced habitat quality [38,71,72]. However, the exact benefits of the hunting reserves have not been scrutinised exhaustively in the international literature, and it is still unclear how widely applicable they are and which species are likely to benefit the most.

On the contrary, the effects of roads have been studied extensively by now [32,35,66,73]. Many studies have shown that the effects of roads on birds are mostly negative, affecting the species in multiple and complex ways [32]. For example, roads can increase mortality through collisions with vehicles, or change birds’ behavior and fitness by increasing the levels of noise pollution [32,35]. Even in the cases where roads are rarely used by cars, they can still have a negative effect [74], by resulting in habitat loss and degradation. Interestingly though, the effect of roads in our study although it was negative it was not large as expected (Table 3). In a sense, this could have been also deduced from the fact that several of the biodiverse wetlands in our dataset are surrounded by a dense network of roads. Larnaca Salt Lake for example, the second most biodiverse wetland in our study, is dissected by a major road, which leads to the island’s largest international airport few hundred meters nearby. Other wetlands, such as Oroklini Lake and Paralimni Lake also neighbour roads with high-traffic flow.

To conclude, our results provide evidence for the idea that under the appropriate conditions, certain artificial wetlands can support diverse communities, and therefore their conservation value should not be discounted [5,75]. Although it is reasonable that priority should be given to natural wetlands [29,64,76], as they generally tend to support more species and more individuals [29,76], conservation actions can be complemented through the protection of existing biodiverse artificial wetlands [23,27]. Prohibition of hunting in the immediate surrounding area is likely to have a positive effect. Lastly, building a dense network of roads near wetlands should be avoided, because despite a level of tolerance that some wetland species may show–an interesting pattern that needs to be examined further in the future–roads can have a negative effect on birds [70].

## Supporting information

S1 Fig The distributions of the overall beta diversities and for each type of wetlands, measured using the Bray-Curtis dissimilarity index. (TIFF)

S1 Table Results of the two-tailed t-tests comparing species richness and diversity between natural and artificial wetlands using the data from the monthly bird surveys of 2009, 2010, and 2011. (DOCX)

S2 Table The list of species accounting for 90% of the dissimilarity in species composition between artificial and natural wetlands in 2009, when abundances are used. For each species, the average and the relative average abundances in each wetland type are shown, along with the cumulative contribution to the Bray-Curtis dissimilarity index.
(DOCX)

S3 Table The list of species accounting for 90% of the dissimilarity in species composition between artificial and natural wetlands in 2010, when abundances are used. For each species, the average and the relative average abundances in each wetland type are shown, along with the cumulative contribution to the Bray-Curtis dissimilarity index.
(DOCX)

S4 Table List of all the regression models with a ΔAICc of less than 2 for species richness and diversity. For each of the model the unstandardized regression coefficients are shown, along with the model’s log-likelihood (logLik), AICc, ΔAICc, and weight.
(DOCX)
