# Supplementary figures and images for: The importance of artificial wetlands for birds: A case study from Cyprus
Source: PLoS One. 2018 May 10;13(5):e0197286. doi: 10.1371/journal.pone.0197286 (PMC5945047; doi:10.1371/journal.pone.0197286)

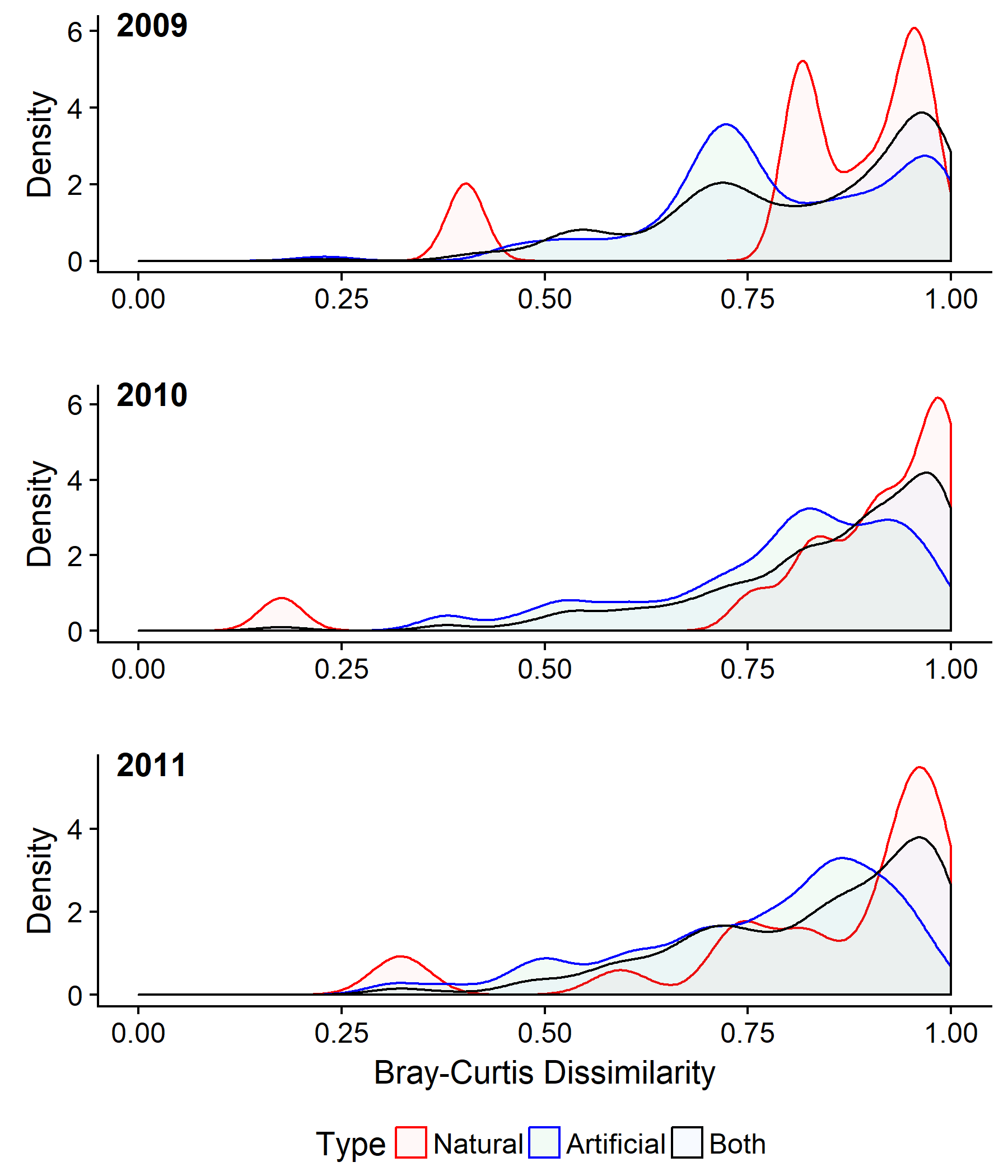

Supplement: S1 Fig — (TIFF) [file pone.0197286.s001.tiff]
