# Supplementary material for: The importance of artificial wetlands for birds: A case study from Cyprus
Source: PLoS One. 2018 May 10;13(5):e0197286. doi: 10.1371/journal.pone.0197286 (PMC5945047; doi:10.1371/journal.pone.0197286)
Supplement: S1 Table — (DOCX) [file pone.0197286.s002.docx]

**S1 Table. Results of the two-tailed t-tests comparing species richness and diversity between natural and artificial wetlands using the data from the monthly bird surveys of 2009, 2010, and 2011.**

| Year | t | df | p-value |
| --- | --- | --- | --- |
| Species Richness | | | |
| 2009 | 1.841 | 6.187 | 0.114 |
| 2010 | 1.209 | 7.180 | 0.265 |
| 2011 | 2.207 | 7.402 | 0.061 |
| Species Diversity | | | |
| 2009 | 1.732 | 11.571 | 0.110 |
| 2010 | 1.080 | 10.595 | 0.304 |
| 2011 | 2.158 | 11.480 | 0.053 |
