# Supplementary material for: The importance of artificial wetlands for birds: A case study from Cyprus
Source: PLoS One. 2018 May 10;13(5):e0197286. doi: 10.1371/journal.pone.0197286 (PMC5945047; doi:10.1371/journal.pone.0197286)
Supplement: S2 Table — For each species, the average and the relative average abundances in each wetland type are shown, along with the cumulative contribution to the Bray-Curtis dissimilarity index. (DOCX) [file pone.0197286.s003.docx]

**S2 Table. The list of species accounting for 90% of the dissimilarity in species composition between artificial and natural wetlands in 2009, when abundances are used. For each species, the average and the relative average abundances in each wetland type are shown, along with the cumulative contribution to the Bray-Curtis dissimilarity index.**

| Species | Average abundance | | Relative average  abundance | | Cumulative  Contribution |
| --- | --- | --- | --- | --- | --- |
|  | Artificial | Natural | Artificial | Natural |  |
| *Phoenicopterus roseus* | 7.54 | 2715.20 | 0.00 | 1.00 | 0.19 |
| *Fulica atra* | 233.23 | 391.00 | 0.37 | 0.63 | 0.30 |
| *Larus ridibundus* | 177.85 | 725.40 | 0.20 | 0.80 | 0.40 |
| *Anas crecca* | 223.69 | 165.40 | 0.57 | 0.43 | 0.45 |
| *Charadrius alexandrinus* | 29.31 | 491.80 | 0.06 | 0.94 | 0.50 |
| *Spatula clypeata* | 312.69 | 116.80 | 0.73 | 0.27 | 0.53 |
| *Calidris minuta* | 60.46 | 428.20 | 0.12 | 0.88 | 0.56 |
| *Tachybaptus ruficollis* | 73.31 | 52.20 | 0.58 | 0.42 | 0.59 |
| *Larus cachinnans* | 36.69 | 228.40 | 0.14 | 0.86 | 0.62 |
| *Anas platyrhynchos* | 142.69 | 102.00 | 0.58 | 0.42 | 0.65 |
| *Himantopus himantopus* | 18.31 | 143.20 | 0.11 | 0.89 | 0.68 |
| *Calidris pugnax* | 76.85 | 78.40 | 0.49 | 0.51 | 0.71 |
| *Vanellus spinosus* | 34.92 | 43.80 | 0.44 | 0.56 | 0.73 |
| *Larus michahellis* | 34.69 | 182.40 | 0.16 | 0.84 | 0.75 |
| *Egretta garzetta* | 15.62 | 37.40 | 0.29 | 0.71 | 0.77 |
| *Spatula querquedula* | 5.31 | 269.60 | 0.02 | 0.98 | 0.78 |
| *Ardea cinerea* | 31.08 | 100.80 | 0.24 | 0.76 | 0.80 |
| *Gallinula chloropus* | 20.08 | 36.40 | 0.36 | 0.64 | 0.81 |
| *Tadorna tadorna* | 1.54 | 183.00 | 0.01 | 0.99 | 0.83 |
| *Platalea leucorodia* | 2.69 | 17.40 | 0.13 | 0.87 | 0.84 |
| *Calidris alpina* | 9.69 | 121.40 | 0.07 | 0.93 | 0.85 |
| *Bubulcus ibis* | 24.15 | 4.60 | 0.84 | 0.16 | 0.86 |
| *Burhinus oedicnemus* | 0.85 | 126.80 | 0.01 | 0.99 | 0.87 |
| *Tringa glareola* | 10.23 | 61.40 | 0.14 | 0.86 | 0.88 |
| *Phalacrocorax carbo* | 23.92 | 0.80 | 0.97 | 0.03 | 0.89 |
