# Supplementary material for: The importance of artificial wetlands for birds: A case study from Cyprus
Source: PLoS One. 2018 May 10;13(5):e0197286. doi: 10.1371/journal.pone.0197286 (PMC5945047; doi:10.1371/journal.pone.0197286)
Supplement: S3 Table — For each species, the average and the relative average abundances in each wetland type are shown, along with the cumulative contribution to the Bray-Curtis dissimilarity index. (DOCX) [file pone.0197286.s004.docx]

**S3 Table. The list of species accounting for 90% of the dissimilarity in species composition between artificial and natural wetlands in 2010, when abundances are used. For each species, the average and the relative average abundances in each wetland type are shown, along with the cumulative contribution to the Bray-Curtis dissimilarity index.**

| Species | Average abundance | | Relative average  abundance | | Cumulative  Contribution |
| --- | --- | --- | --- | --- | --- |
|  | Artificial | Natural | Artificial | Natural |  |
| *Phoenicopterus roseus* | 69.15 | 4107.67 | 0.02 | 0.98 | 0.22 |
| *Fulica atra* | 292.77 | 397.83 | 0.42 | 0.58 | 0.36 |
| *Calidris pugnax* | 18.08 | 338.83 | 0.05 | 0.95 | 0.40 |
| *Tachybaptus ruficollis* | 119.38 | 44.67 | 0.73 | 0.27 | 0.45 |
| *Anas platyrhynchos* | 198.31 | 76.33 | 0.72 | 0.28 | 0.49 |
| *Charadrius alexandrinus* | 12.08 | 446.17 | 0.03 | 0.97 | 0.53 |
| *Larus cachinnans* | 108.54 | 53.83 | 0.67 | 0.33 | 0.56 |
| *Spatula clypeata* | 316.31 | 136.83 | 0.70 | 0.30 | 0.60 |
| *Anas crecca* | 210.38 | 105.50 | 0.67 | 0.33 | 0.63 |
| *Larus ridibundus* | 146.69 | 422.17 | 0.26 | 0.74 | 0.67 |
| *Vanellus spinosus* | 58.08 | 66.33 | 0.47 | 0.53 | 0.69 |
| *Himantopus himantopus* | 24.62 | 181.83 | 0.12 | 0.88 | 0.72 |
| *Egretta garzetta* | 28.38 | 61.17 | 0.32 | 0.68 | 0.74 |
| *Gallinula chloropus* | 54.38 | 35.33 | 0.61 | 0.39 | 0.76 |
| *Bubulcus ibis* | 60.46 | 12.33 | 0.83 | 0.17 | 0.78 |
| *Ardea cinerea* | 32.92 | 84.17 | 0.28 | 0.72 | 0.80 |
| *Calidris minuta* | 12.46 | 245.67 | 0.05 | 0.95 | 0.82 |
| *Phalacrocorax carbo* | 22.15 | 34.50 | 0.39 | 0.61 | 0.84 |
| *Nycticorax nycticorax* | 22.31 | 27.83 | 0.44 | 0.56 | 0.85 |
| *Larus michahellis* | 27.77 | 103.83 | 0.21 | 0.79 | 0.86 |
| *Tadorna tadorna* | 6.62 | 213.83 | 0.03 | 0.97 | 0.88 |
| *Ardea purpurea* | 15.31 | 5.17 | 0.75 | 0.25 | 0.89 |
| *Aythya nyroca* | 9.31 | 21.83 | 0.30 | 0.70 | 0.89 |
