# Supplementary material for: The importance of artificial wetlands for birds: A case study from Cyprus
Source: PLoS One. 2018 May 10;13(5):e0197286. doi: 10.1371/journal.pone.0197286 (PMC5945047; doi:10.1371/journal.pone.0197286)
Supplement: S4 Table — For each of the model the unstandardized regression coefficients are shown, along with the model’s log-likelihood (logLik), AICc, ΔAICc, and weight. (DOCX) [file pone.0197286.s005.docx]

**S4 Table. List of all the regression models with a ΔAICc of less than 2 for species richness and diversity. For each of the model the unstandardized regression coefficients are shown, along with the model’s log-likelihood (logLik), AICc, ΔAICc, and weight.**

| Depth | Hunting reserves (%) | Area  (km^2^; log) | Road Density (km/km^2^; log) | logLik | AICc | ΔAICc | weight |
| --- | --- | --- | --- | --- | --- | --- | --- |
| Species Richness (R^2^ = 0.70) | | | | | | | |
| + | 57.38 | 4.92 |  | -74.30 | 163.21 | 0.00 | 0.72 |
| + | 53.19 | 4.10 | -5.94 | -73.07 | 165.13 | 1.92 | 0.28 |
| Species Diversity (R^2^ = 0.42) | | | | | | | |
| + |  |  | -1.50 | -44.14 | 99.14 | 0.00 | 0.25 |
| + |  |  |  | -45.93 | 99.46 | 0.32 | 0.21 |
| + | 5.14 |  |  | -44.45 | 99.76 | 0.62 | 0.18 |
| + | 4.42 |  | -1.33 | -42.87 | 100.35 | 1.21 | 0.14 |
| + | 5.85 | 0.62 |  | -42.97 | 100.56 | 1.42 | 0.12 |
| + |  | 0.51 |  | -45.08 | 101.02 | 1.88 | 0.10 |
